# Genome Sequence of KP-Rio/2015, a Novel *Klebsiella pneumoniae* (*Podoviridae*) Phage

**DOI:** 10.1128/genomeA.01298-16

**Published:** 2016-12-22

**Authors:** Guilherme L. S. Meira, Fabricio S. Campos, Julia P. Albuquerque, Maulori C. Cabral, Sergio E. L. Fracalanzza, Renata M. Campos, Alane B. Vermelho, Davis F. Ferreira

**Affiliations:** a Institute of Microbiology, Federal University of Rio de Janeiro, Rio de Janeiro, Brazil; b Veterinary Medicine and Agronomy, University of Brasilia, Brasilia, Brazil; c Biomedical Institute, Fluminense Federal University, Rio de Janeiro, Brazil

## Abstract

*Klebsiella pneumoniae* is a pathogen frequently associated with antibiotic-resistant nosocomial infections. Here, we describe the genome of KP-Rio/2015, a novel phage of* K. pneumoniae* belonging to the family *Podoviridae*.

## GENOME ANNOUNCEMENT

*Klebsiella pneumoniae* is ubiquitous in nature and predominately associated with antibiotic-resistant nosocomial infections (1). Carbapenemase-producing *K. pneumoniae* is a highly drug-resistant bacterium in the family *Enterobacteriaceae*. It can easily be spread in hospital settings, provoking deadly systemic infections (1, 2). Bacteriophage-based therapy has been regaining the scientific community’s attention, and this has led to increased research to identify prospect phages from different sources in order to find candidates that will kill multidrug-resistant bacteria (3). Recent *K. pneumoniae* phage discoveries include KP34, SU503, and SU552A (4, 5).

In this work, we describe the complete genome of KP-Rio/2015, a novel *K. pneumoniae* phage belonging to the family *Podoviridae*. The *K. pneumoniae* bacterium was isolated from a human urine sample, and the phage was isolated from urine antibiogram. The phage was concentrated using polyethylene glycol. Phage DNA was extracted by phenol-chloroform, sequenced in an Illumina HiSeq 2000 sequencing platform by GenOne Biotechnologies (Rio de Janeiro, Brazil). A total of 1,282,326 reads of ~150 bp were assembled with Geneious version 8.1.8. The reads were *de novo* assembled, with a mean coverage of 4,416×. A contig of 43,557 nucleotides comprised the full KP-Rio/2015 genome. With a G+C genomic content of 54.1%, we identified 47 open reading frame candidates, 11 regulatory regions, and five repeat regions in the KP-Rio/2015 genome. Predicted proteins encoded by this virus were DNA/RNA polymerases, a helicase, head and tail phage proteins, and hypothetical proteins; most of them exhibit sequence similarity (83.3 to 94.7%) to other subfamily *Autographivirinae* proteins. The genome sequence of KP-Rio/2015 had nucleotide identities with the following *K. pneumoniae* phages: SU552A (81.0%), SU503 (72.2%), F19 (72.1%), KP34 (74.2%), and NTUH-K2044-K1-1 (73.5%). The identity percentages were calculated by alignment of full-genome sequences. High identity with SU552A suggests that KP-Rio/2015 may have the potential to kill multidrug-resistant *K. pneumoniae*. Further experiments are necessary to confirm such an ability.

### Accession number(s).

The GenBank accession number is KX856662.
